# A Novel Method to Detect Partial Shadow Effects in Perovskite-Based Simulated Solar Cell System Faults

**DOI:** 10.3390/mi14040832

**Published:** 2023-04-11

**Authors:** Amir Sharifi Miavaghi, Asghar Esmaeili

**Affiliations:** Faculty of Science, Department of Physics, Urmia University, Urmia 5756151818, Iran; a.esmaeili@urmia.ac.ir

**Keywords:** partial shadow, solar system, support vector machine, classification

## Abstract

When a fault occurs in photovoltaic systems, a human expert should be present at the scene and perform tests to determine the location and type of the fault. In such a situation, in order to maintain the safety of the specialist, protective measures such as shutting down the power plant or isolating the faulty part are usually taken. Given the fact that the equipment and technology of photovoltaic systems are expensive and their efficiency is currently relatively low (about 20%), a complete shutdown of the plant or part of it can be economical, return on investment and achieve profitability. Therefore, as much as possible, efforts should be made to detect and eliminate errors in the shortest possible time without shutting down the power plant. On the other hand, most solar power plants are located in desert areas, which make them difficult to access and visit. In this case, training skilled manpower and the constant presence of an expert on site can be very costly and uneconomical. Also, if these errors are not detected and fixed in time, they can lead to power loss (not using the maximum potential of the panel), device failure and eventually fire. In this research, using fuzzy detection method, a suitable method for detecting the error of partial shadow occurrence in solar cells is presented. Based on the simulation results, the efficiency of the proposed method is confirmed.

## 1. Introduction

According to statistics published in 2018 by the International Energy Agency, 37.5% of carbon dioxide and other greenhouse gases produced by various sources mainly came from fossil fuel power plants [1,2,3]. Due to the destructive effects of greenhouse gases on the environment, such as global warming, surface water pollution, acid rain, etc., extensive measures have been taken to address these problems and reduce their effects.in recent years One of the most effective ways to reduce greenhouse gases from power plants has been the use of clean sources such as solar and wind energy [4,5,6,7,8,9]. Photovoltaic systems convert solar energy directly into electrical energy and do not pollute the environment compared to traditional power generation systems. For this reason, photovoltaic systems are also called “green” or “clean” energy sources. Simultaneously with the increase in problems caused by environmental pollution and also the decrease in fossil energy reserves, the use of photovoltaic systems has increased sharply in recent years. Historically, photovoltaic systems, due to their high cost, were commonly used to provide the electrical power needed by satellites [10,11]. However, with the increasing growth of the semiconductor industry in recent years and the reduction of production costs of semiconductor devices, solar arrays have found wide applications today, one of which is grid-connected photovoltaic systems. The use of electronic power devices causes harmonics in the output voltage of the photovoltaic system, which should be eliminated as much as possible.

In 1998, Edward Owen published a history of harmonics on the power grid. He cites the experience of the city of Hotford in the United States in 1893 as the first problem of harmonic distortion, and that power engineers were justified by the problem of an overheating of an electric motor and its insulation failure. It is worth noting that this engine was manufactured in the factory and shipped well before being shipped to Hartford. The only difference between the factory test conditions and the actual working conditions in Hartford was a 10-mile transmission line. In order to find the cause of this problem, a harmonic analysis was performed on the shape of the current waves and the voltage of the transmission line that fed the motor. The results demonstrated that the engine heats up due to the intensification in the transmission line due to the presence of harmonics [10,12]. 

The power generated by this generator was transmitted through a transmission line to the other side of Hartford, where it was powered by a synchronous motor. The synchronous motor, in turn, acted as a drive for a DC generator that powered the city’s trains. Could. Because the designers were unaware of the possibility of escalation in the transmission line, they also did so. In fact, by calculating the inductance and capacitance of the transmission line and possibly the charge inductance, they observed that at a frequency of about 1600 Hz (the thirteenth harmonic of the main frequency) is created in the resonant line. The voltage waveforms of the power plant generator and synchronous motor had significant harmonic components. Perhaps the most interesting aspect of this research is how they were able to get started with a lot of tools and equipment. They did not have access to modern measuring equipment such as an oscilloscope or harmonic meter. In 1893, it was not even possible to access a good voltmeter. Osilographs had not yet been invented, and the only device that could be used as the waveform, which sampled the waveform point-by-point through the regular cutting and connecting of a tab. The wavefinder detector used a zero galvanometer instead of a deflection galvanometer, which meant that a bridge had to be balanced everywhere. However, they managed to record the waveforms and perform Fourier analysis on it. Waves do. According to available reports, the calculation of each of the Fourier coefficients took one hour. It is worth noting that the waveguide commutator recorded information at a frequency of 4500 Hz, which for the base frequency of 125 Hz, 36 samples will be obtained per cycle. Nowadays, harmonic distortions can be measured in real time by using digital harmonic meters and by using fast discrete Fourier transform algorithms. In general, voltage and current wave disturbances are expressed in terms of harmonic frequencies, which are exact coefficients of the original frequency. Harmonic currents as well as factors, their creation, standards, measurement, simulation and elimination. Since then, the unexpected increase in the number and nominal values of solid-state elements to control power systems and equipment has caused harmonic problems inside and outside the power system. Harmonic correction is a costly and uncommon method, and usually applied according to the theory of “better prevention than cure”. More thinking and investing is endertaken in the design process. However, prevention methods are also costly and their optimization, which is one of the basic stages of design, relies heavily on theoretical estimates. Accurate estimation of harmonics requires an understanding of two separate and completely interdependent issues. The first is the nonlinear characteristic effects of voltage-current on some parts of the system, or in other words, the presence of harmonic sources in the power system. In this regard, the main problem is the accurate knowledge of harmonic sources. The second issue is to extract the appropriate harmonic model for the dominant linear components in the system in order to calculate the harmonic load propagation resulting from their connection in the network. The main problem here is the lack of information about how the system’s loads are combined and the amount of damping at harmonic frequencies. Other obstacles to accurately estimate harmonics are the large number of nonlinear characteristics distributed in the system, phase duality, load variation, and so on. Also, proper and planned use of these resources can reduce losses, improve voltage profiles and increase security in power networks [13].

One of the most efficient and widely used power plants based on renewable energy sources are solar power plants (photovoltaic systems). The technology of these systems are advancing day by day and since 2010, the cost of generating electricity by these systems has been reduced by more than 70% [14]. According to statistics released by the International Energy Agency, the amount of electricity produced by solar power plants in 2018 has increased by more than 40% compared to the previous year and is expected to reach 17% by 2030 compared to the current level [15]. At present, the total production capacity of solar power plants worldwide is about 460 terawatt hours (TWh), which is a total of two percent of the total electricity consumption [16]. Due to the geographical location of our country (Iran), the intensity of good sunlight in most parts of the country and the many benefits of these power plants, paying attention to these power plants and their expansion is extremely important [17].

**Figure 1:**
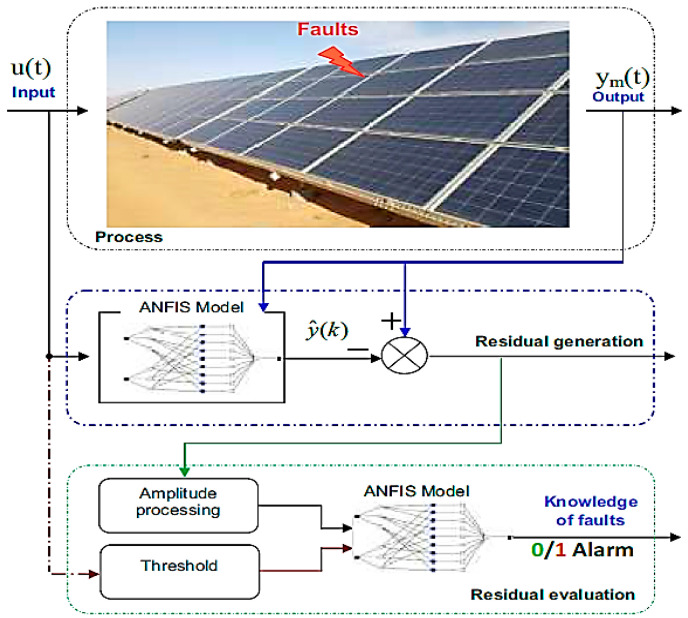
Structure of the method presented to model the behavior of the panel in different weather conditions [21].

However, due to the variability of solar intensity and other atmospheric variables involved in the production capacity of photovoltaic systems, the use of these systems faces limitations and restrictions, and therefore in recent years, many studies on the system of Photovoltaics have been performed. In these studies, various aspects of photovoltaic systems such as tracking the maximum power point, predicting the amount of power generated by the power plant, optimal location of these power plants, estimating equivalent circuit parameters, reactive power control of these systems and other issues are investigated [18,19,20,21]. One of the important issues regarding photovoltaic systems is the occurrence of various errors such as module short-circuit failure and diode disconnection, which can reduce the efficiency of these systems and endanger their security. If these errors are not identified and fixed in time, they can lead to power loss (not using the maximum potential of the panel), device failure, and eventually fire [14,22]. In recent years, various studies have been conducted on the timely and accurate detection of errors in photovoltaic systems and various methods have been proposed. In all these methods, the current, voltage and output power of the sampled panel are compared with its normal state and the type of error is determined. For example, in reference number [23], the behavior of the photovoltaic panel is first modeled by the adaptive neural-fuzzy inference system (ANFIS) to measure the amount of current, voltage and power of the panel for different atmospheric inputs (temperature and intensity of sunlight) To be estimated. Data collected from a solar power plant in Algiers is used to train Anfis. ANFIS is an adaptive system based on fuzzy inference network that has the ability to learn the approximation of nonlinear functions. This interface is trained only for normal panel mode and can model the panel behavior in normal mode. The structure of the method presented in Figure 1 is shown. In this figure, the ANFIS Model (middle row) is used to model the behavior of the panel in different weather conditions. At any given time, the intensity of sunlight and temperature is sampled and given to the ANFIS Model to determine its normal output ($\hat{y}$). The normal state of that atmospheric condition ($\hat{y}$) is compared with the actual output and the difference between these values is $\Delta P=P_{healthy}-P_{real}$, $\Delta V=V_{healthy}-V_{real}$ and $\Delta I=I_{healthy}-I_{real}$ is calculated. After calculating Δ*P*, Δ*V* and Δ*I*, these values are used as the input of the second infin (bottom row) to identify the type of error.

**Figure 2:**
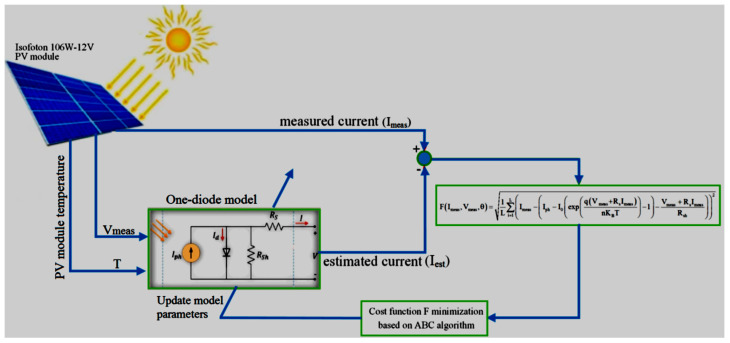
Calculation of equivalent circuit parameters [22].

In Figure 1, to analyze the location of scattered production resources in a simple way, it may be necessary to consider over a billion states to find the optimal location and size. Due to the time limit, we need to use optimization algorithms to find the answer. Therefore, optimization algorithms are used in location problems. The bee colony algorithm has become widespread in optimization today and usually most of them use this algorithm to solve optimization problems. In reference number [24], the equivalent circuit is used to model the photovoltaic panel. Equivalent parameters were obtained using the artificial bee cloning algorithm. In this method, a single diode model is used (Figure 2). In this method, for different atmospheric conditions, the outputs are calculated and compared with the actual value, and the remaining values, i.e., Δ*P*, Δ*V* and Δ*I* are calculated. In the next step, based on a series of predetermined criteria, the type of error is determined.

Various studies have been done in this field, but the accuracy of error detection is still not high. Some examples of disadvantages of the previous methods are:

## 2. Simulation

Standard mesoscopic PSC structure made of FTO coated glass, a layer of TiO_2_ (as an electron transporter and scaffold), a perovskite absorbent layer, a cavity transfer layer and a gold back electrode or silver is formed so that this electrode can be replaced with other compounds such as carbon.

The most common material used as a cavity transporter in PSCs is the Spiro-OMeTAD polymer compound. The characteristics of this compound such as suitable glass transition temperature, solubility, ionization potential and transparency in the visible spectrum range make this compound a suitable option for these applications. Additives such as lithium salt (LiTFSI) and tert-butyl pyridine can be used with this compound to increase the mobility of holes.

Theoretically, the maximum efficiency of a pericardial solar cell (MAPbI3) with a thickness of one micron is 26%, which is much higher than the efficiency of a GaAs solar cell with a similar thickness. This indicates the high commercialization capacity of these cells. PSCs have performed well for up to 1000 h so far, but these results are still not enough to commercialize the cells. The lifetime of these cells should be more than 20 years. This will be possible by accurately understanding the mechanism of cell function and the factors affecting it, the proper formulation of perovskite inks for coating and the use of new materials chemistry and technologies to encapsulate cells. Reducing the cost of materials and manufacturing processes is another important challenge in commercializing these cells [25]. 

**Figure 3:**
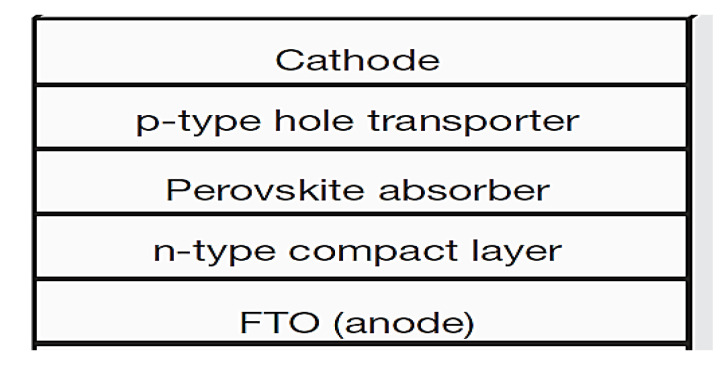
Solar cell structure examined in this paper.

**Figure 4:**
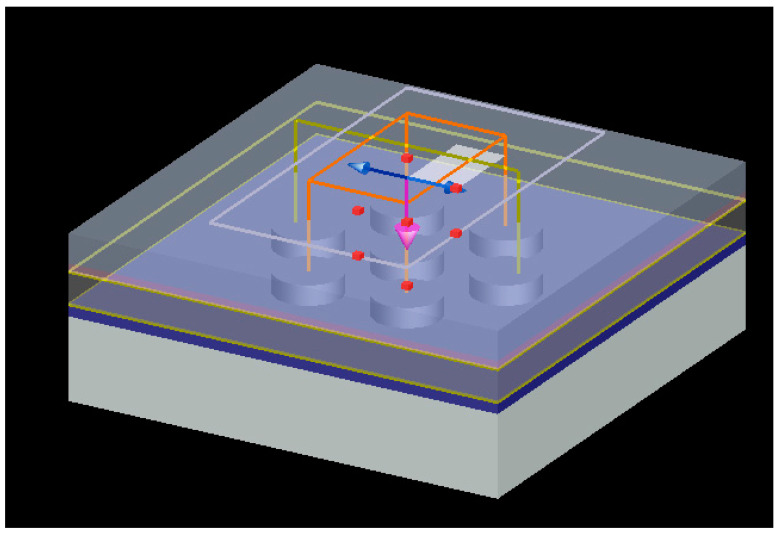
PSC structure designed with nanoparticles.

The simulation results of Perovskite solar cell (PSCs) structure based on graphene nanowires are investigated. Based on the information and specifications of this data sheet, the material selected for the structure of the solar cell is shown in Figure 3 The shape is such that first an anode layer is provided for this purpose; transparent semiconductor layers used as anodes and cathodes in solar cells come in a variety of forms, the most common of which is fluorine-doped tin oxide (FTO). In this paper, FTO is used as the anode. The next layer is a compact layer of type n, which is used as a free electron absorber. The next layer is the perovskite adsorbent layer. This material has a very effective crystalline structure that can process energy needs but can be problematic when exposed to environmental hazards such as moisture. Researchers have been able to increase the efficiency of perovskite-based solar cells, but this substance has more potential than previously obtained. The next layer is used to transfer holes, which is type p. Finally, the last layer is the cathode, which in this paper is also selected from graphene nanowire. Figure 4 shows the implementation structure in Lumerical software. For more accuracy, the simulation was first performed on silicon solar cells and then compared with a prosciutto-based graphene nanowire solar cell. Lead-iodide methyl ammonium is an ambipolar semiconductor compound that can transfer both electrons and cavities to the corresponding collecting electrodes. This is because PSCs can work even in the absence of a hole conductor or electron conductor. The unique properties of lead iodide perovskites, as well as its low cost and simplicity of processing, make it highly suitable for use in high-efficiency, low-cost third-generation solar cells [26].

**Figure 5:**
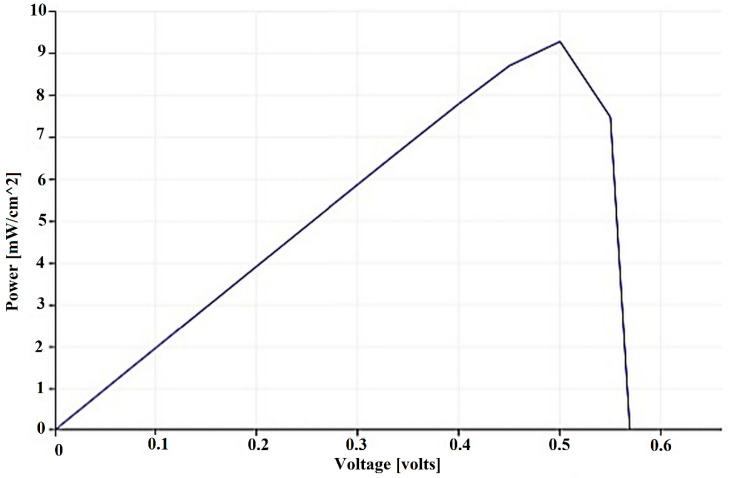
Voltage-based output power curve for PSC designed with gaphene nanoribbon.

Figure 5 shows the voltage-based output power curve for a PSC designed with gaphene nanoribbon. Based on the open circuit voltage for the PSC designed with gaphene nanoribbon, it has reached a voltage of 0.56 volts and the maximum power at a voltage of 0.5 volts is 9 mW. In this case, the perovskite material not only increased the efficiency of the solar cell, but also achieved greater persistence. It also retains 80% of its original energy conversion ability even after 1000 h of continuous light. This solar cell is based on a recombinant layer of perovskite with a wide energy gap called Apex Flex, which is able to withstand heat, light and performance tests and simultaneously produce high and reliable voltage [27]. One of the main layers of a PSC is the electron transfer layer, which is responsible for moving electrons from the perovskite layer to the tin oxide-conducting glass with doped fluorine. The composition used in this layer can affect electronic parameters such as current density, charge coefficient and open circuit voltage, and changing these parameters can affect the efficiency and stability of solar cells. Figure 5 shows the voltage-based current density curve for a PSC designed with nano-graphene. The gaphene nanoribbon reaches 1.89 mA.

### 2.1. Detecting Partial Shadow as Error

**Figure 6:**
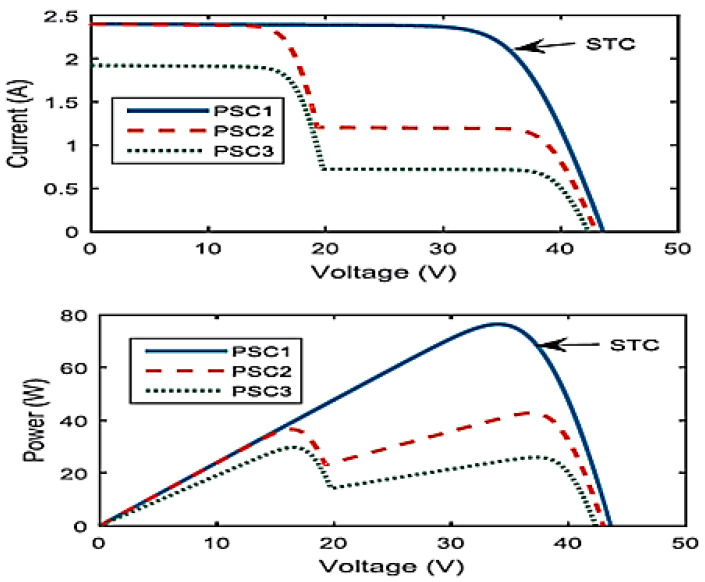
Different partial shading patterns, Voltage-current and voltage-power curves (Vikram Solar ELDORA-37 modules) [28].

In most of the proposed methods to detect the type of error in photovoltaic systems, changes in the amount of output power and changes in voltage-current curve have been used to determine the type of error. The effect that the occurrence of a small shadow on the power and voltage-current curve is very similar in practice to the effects that different errors have on the power and voltage-current curve. Figure 6 shows the voltage-current and voltage-power curves under different partial shadows. In this figure, the PSCs represent certain conditions of partial shadowing. It can be seen that the presence of a slight shadow caused a change in voltage, current, and output power of the panel.

For an efficient Photovoltaic (PV) system, tracking of true maximum power point (MPP) is essential. Therefore, the maximum power point tracking (MPPT) controller is mandatory for harvesting maximum power from the solar panel. In this paper, the proposed ABC-PO algorithm is implemented in MATLAB/Simulink model, and it is compared with different MPPT algorithms such as P&O, Incremental conductance (INC) and ABC (Figure 3). Table 1 also lists some common faults in the photovoltaic panel. Mode one (PF1) is related to the normal state where there were no errors in the system [29].

### 2.2. Use a Series of Parameters for All Weather Conditions

According to studies conducted in recent years, the equivalent circuit parameters of a photovoltaic panel change significantly with changing environmental conditions (temperature and radiation intensity). This issue has been thoroughly investigated in reference [21]. Therefore besides the studies that use the equivalent circuit to calculate the residual (Δ*P*, Δ*V and* Δ*I*), it can be to detect a partial shadow as an error, also (Δ*P*, Δ*V and* Δ*I*) have difficulty in identifying other types of errors, because in these studies for all conditions atmosphere is a series of fixed parameters (for radiation intensity G = 1000 and T = 25 °C). In order to increase the accuracy, the value of the equivalent circuit parameters must be updated for each weather condition.

### 2.3. Using Raw Data Such as (*Δ*P, *Δ*V and *Δ*I) or Voltage-Current Diagrams, etc. as Input

According to the studies conducted in the field of pattern recognition using intelligent methods, the type of input and its volume have a high impact on the accuracy of detection, with the help of which new features of the voltage-current curve and changes can be extracted and to improve the accuracy Diagnosis is used.

Due to the importance of online fault detection and its type detection in photovoltaic systems, in this study a new method has been proposed for this purpose. The proposed method will consist of three parts: the part of detecting the occurrence of perturbation (partial shadow and error), the part of separating partial shadow from other errors and the part of detecting the type of error.

Part One: Disturbance Detection (Partial Shadow and Error)

In this method, the voltage and current of the panel are sampled and converted to per unit value.

(1) $$v_{PV}^{pu}\left(kT_{s}\right)=\frac{v_{PV}\left(kT_{s}\right)}{V_{OC}}$$

(2) $$i_{PV}^{pu}\left(kT_{s}\right)=\frac{i_{PV}\left(kT_{s}\right)}{I_{SC}}$$

(3) $$p_{PV}^{pu}\left(kT_{s}\right)=v_{PV}^{pu}\left(kT_{s}\right)\times i_{PV}^{pu}\left(kT_{s}\right)$$

In the above Equations (1)–(3), k represents the sampling step and T_s_ represents the sampling rate. The value of *T_s_* should be the smallest number must be selected. *T_s_* = 1 ms is a good value. When an error or partial shadow occurs, perturbations occur in the panel behavior that introduce new components into the system signals. These components are called added or superimposed components (SI) [30]. The output power of the panel in the event of an error or partial shadow can be written based on the following components:

(4) $$p_{PV,F}^{pu}\left(kT_{s}\right)=p_{PV,N}^{pu}\left(kT_{s}\right)+p_{PV,SI}^{pu}\left(kT_{s}\right)$$

In Equation (4), $p_{PV,N}^{pu}\left(kT_{s}\right)$ indicates power of the panel in normal mode, $p_{PV,SI}^{pu}\left(kT_{s}\right)$ mounted component and $p_{PV,F}^{pu}\left(kT_{s}\right)$ power in the whole panel.

The mounted component can be calculated by the following equation:

(5) $$p_{PV,SI}^{pu}\left(kT_{s}\right)=p_{PV}^{pu}\left(kT_{s}\right)-p_{PV,SI}^{pu}\left(kT_{s}-T_{d}\right)$$

In the above relation (5), *T_d_* indicates the time delay. The mounted component $p_{PV,SI}^{pu}\left(kT_{s}\right)$ has a small value. Therefore, this component can be multiplied by a constant value to make its value more significant:

(6) $$p_{PV,SI}^{{}^{\prime }pu}\left(kT_{s}\right)=K_{amp}\times \left|p_{PV,SI}^{pu}\left(kT_{s}\right)\right|$$

*K_amp_* is a large fixed number.

In Equation (6) If the value of $p_{PV,SI}^{{}^{\prime }pu}\left(kT_{s}\right)>\varepsilon$, an error is detected. In this method, ε is the error detection threshold. The value of $p_{PV,SI}^{{}^{\prime }pu}\left(kT_{s}\right)$ for normal climate change is close to zero. However, when a partial shadow is formed or an error occurs in the system, its value will be large and with these relationships, their occurrence can be detected. 

## 3. Partial Shadow Separation from Other Errors

In the next step, after the occurrence of perturbation (partial shadow and error), we need another feature to separate the partial shadow from the error. According to the studies and surveys, it was observed that the time interval of power changes for partial shadow mode and error is different from each other. When perturbation occurs, the amount of power generation decreases over a period of ∆T. In these cases, the mounted component first increases during the period ∆*T*1, then decreases during the period ∆*T*2.

When an error occurs, the mounted component quickly reaches the maximum value and reaches zero over a longer period of time (∆*T*1 < ∆*T*2).

When a partial shadow occurs, the mounted component reaches a maximum value over a longer period of time, and quickly reaches zero value (∆*T*1 > ∆*T*2).

This panel behavior is due to the presence of a small leakage capacitor parallel to the Rsh resistor [31]. The power changes in the error mode are similar to the view function and in the partial shadow mode are similar to the sigmoid function. Depending on the length of the periods of increase and decrease of the mounted component, the slope of the component in these periods will be different. If we calculate the slope of the component near the maximum point of the mounted component in a window with interval *N*:

(7) $$SLS=\left|\frac{p_{PV,SI}^{{}^{\prime }pu}\left(N_{m}\right)-p_{PV,SI}^{{}^{\prime }pu}\left(N_{0}\right)}{\left(N-1\right)/2\times T_{s}}\right|$$

(8) $$SRS=\left|\frac{p_{PV,SI}^{{}^{\prime }pu}\left(N\right)-p_{PV,SI}^{{}^{\prime }pu}\left(N_{m}\right)}{\left(N-1\right)/2\times T_{s}}\right|$$

In the above relations (7) and (8), *SLS* is the left slope and *SRS* is the right slope of the maximum mounted component point. *N*_0_ also indicates the starting point of the window and *N_m_* indicates the midpoint of the window. If *SLS* > *SRS*, then the perturbation is an error, and if *SLS* < *SRS*, then the perturbation is a partial shadow.

### Line Type Recognition

Once the disturbance is determined to be an error, the type of error must be identified. To do this, the shape properties of the voltage-current curve will be used as the classifier input. Each type of error has its own characteristics that help the classifier to more accurately identify the type of error. Also, in the proposed method, there is no partial shadow in the third part, which, due to its similarity to other errors, reduces the accuracy of the classifier. The following features will be used as effective features:

Feature 1: Area below the voltage-current curve area
  (9) $$F_{1}=S=\overset{V_{OC}}{\underset{0}{\int }}V\left(I\right)dI$$
Second feature: Short circuit current
  (10) $$F_{2}=I_{SC}$$
Third feature: Open circuit voltage
  (11) $$F_{3}=V_{OC}$$
Fourth feature: Maximum power point
  (12) $$F_{4}=max\left(V\times I\right)$$
Feature 5: Maximum power point voltage
  (13) $$F_{5}=V_{MPP}$$
Feature 6: Maximum power point current
  (14) $$F_{6}=I_{MPP}$$
Feature 7: Slope near open circuit point
  (15) $$F_{7}=\frac{dI}{dV}|V_{OC}$$
Feature 8: Slope at the midpoint of the open circuit point and the maximum power point
  (16) $$F_{8}=\frac{dI}{dV}|mid1$$
Ninth feature: Slope at the point of maximum power
  (17) $$F_{9}=\frac{dI}{dV}|MPP$$
Feature 10: Slope at the short circuit point
  (18) $$F_{10}=\frac{dI}{dV}|IS$$
Feature 11: Slope at the midpoint of the open circuit point and the maximum power point
  (19) $$F_{11}=\frac{dI}{dV}|mid2$$
Feature Twelve: Accumulation factor
  (20) $$F_{12}=\frac{F_{5}\times F_{6}}{F_{2}\times F_{3}}$$

According to the Equations (9)–(20) and feature extraction, the features are classified. The superiority of the fuzzy error detector over other classifiers such as neural networks and fuzzy systems has been proven in various references [32,33]. 

When a fault occurs in photovoltaic systems, a human expert should be present at the scene and perform tests to determine the location and type of fault. In such a situation, in order to maintain the safety of the specialist, protective measures such as shutting down the power plant or isolating the faulty part are usually taken. Given that the equipment and technology of photovoltaic systems are expensive, and their efficiency is currently relatively low (about 20%), a complete shutdown of the plant or part of it can be, economically, a return on investment and achieve profitability, but it may be very unpleasant and undesirable. Therefore, as much as possible, efforts should be made to detect and eliminate errors in the shortest possible time without shutting down the power plant. On the other hand, most solar power plants are located in desert areas, which make them difficult to access and visit. In this case, training skilled manpower and the constant presence of an expert on site can be very costly and uneconomical. Also, if these errors are not detected and fixed in time, they can lead to power loss (not using the maximum potential of the panel), device failure, and eventually fire.

Therefore, it is possible to investigate the occurrence of errors in photovoltaic systems from four different aspects:

1. Complete shutdown or part of the power plant for repairs
2. Far from the availability of solar power plants and high cost to train skilled manpower in order to be present on site
3. Power loss
4. Device failure, fire and loss of expensive equipment

In view of the above, it can be seen that it is necessary to provide an accurate method that can automatically detect the occurrence of the error and determine its type. The purpose of this study is to provide an accurate and automatic method to detect the occurrence of errors and detect its type in the shortest time. The proposed method also provides a solution to easily distinguish the occurrence of partial shadows from real errors with high accuracy, without the need for training data.

## 4. Fuzzy Detection Matching

**Figure 7:**
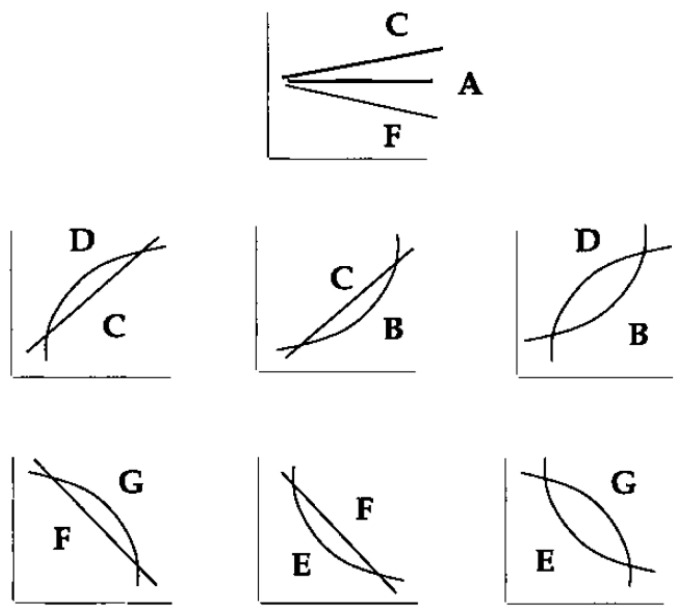
Similarity or dissimilarity of fuzzy species between patterns.

In 1987, Kramer introduced an error detection approach for a chemical process with error patterns and optimal patterns, and showed that the model behaves much more steadily than non-Boolean techniques than Boolean techniques, while the non-Boolean fault detection method is very resistant to noise. In the process of converting recorded data into geometric shapes, many more important features are omitted and reduce accuracy. Of course, in some cases, accuracy will be unnecessary, and for this purpose, a balance between speed, interest and transparency in logical reasoning and other useful cases must be considered. The scope of the fuzzy region has gained many applications in the field of events. Knowledge of how fuzzy logic works for fitting using basic geometric patterns as the event reference language is shown in Figure 7. In this form, there is a smooth overlap between the boundaries of the P elements. This ultimately results in a fuzzy protrusion in P-curved patterns, as the primary language of events. Thus, events naturally focus on fuzzy behavior [34].

The mapping process compares events in several different ways. Which include:

1. Qualitative form of events or in other words the order of *p_i_* patterns
2. The length of the *p_i_* patterns
3. Amplitude changes of *p_i_* patterns

### 4.1. Estimation of Similarity Measurement Using Fuzzy Matching

In this approach, three types of measurements are examined:

1. Matching the similarities between the two patterns
2. Measuring the similarity between two trends or events
3. Assess the overall similarity between the two error scenarios

It is possible to measure similarity; different applications use different methods. In this section, we will deal with two types of similarities, which are simple similarity and similarity with the most weight.

If we call the set of seven basic geometric shapes A to G. They will all be related in relative similarity. For this reason, one of the ways to identify events with more than two strings is to differentiate the *p_i_s*. For example, the symbols B and C are closer to each other in terms of similarity than the two symbols B and E so that we can determine the similarity of all the original patterns to each other. 

Table 2 considered that the horizontal and vertical dimensions (*i*, *j*) indicate the symbols and the numerical values between zero and one degree of relative similarity of each symbol to each other.

The symbol *S* (*p_i_ p_j_*) indicates the relative similarity of the two symbols *p_i_*, *p_j_* in the interval [0,1]. Of course, in the case of similarity matrices, some symbols, such as D and G, behave quite differently, which is set to zero in the table.

The similarity can be considered as a scientific rule. To identify an unknown event and describe it with primary strings would require considering errors in transformations with the highest and lowest similarity criteria. The lowest number of error conversions in the derivative between the two symbols x and y are: Substitute *T_s_*, embedded *T_i_* and omitted *T_D_* are defined as the conversion error grammar [34].

### 4.2. Temporal Synchronization of Segments

**Figure 8:**
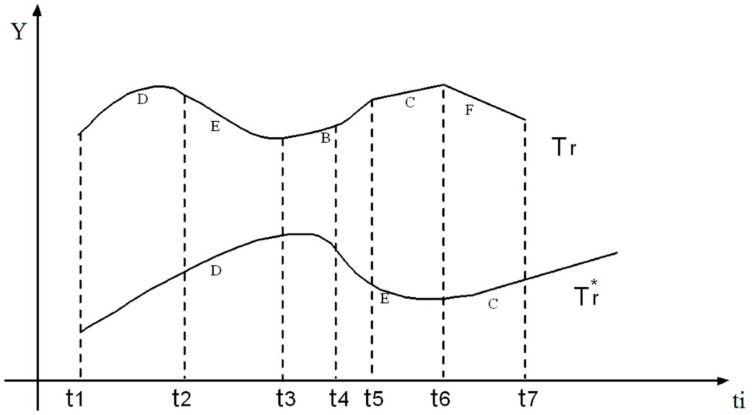
Temporal synchronization of segments.

In order to achieve the appropriate similarity measurement, it is necessary to match the segments in terms of time. That is, the defined time for both segments obtained in the extraction algorithm must be the same and have a logical time interval so that the measurement algorithm can be performed with high accuracy. In synchronization, the minimum and maximum lengths of the segments are calculated by considering the initial time t_i_ and the end time t_f_. This is formed in a new time period in Figure 8. 

For multivariate processes, the overall similarity measurement between the two scenarios must be considered in all power-voltage curves. Fuzzy AND is performed using the min operator, and this is because we need the least differences in the classification of errors. If n is the number of sensors. The similarity or CI measure indicates the probability of a specific error occurring with CI = min {s_1_, s_2_,…, s_k_} where s_k_ is the similarity measurement between the k trend of the power-voltage curve in two scenarios. This method will be resistant to many special aspects such as domain normalization, penalties for large domain differences, adaptation to a common time frame, and the use of the min operator to measure multivariate similarity.

## 5. Simulation

In this section, the results of “simulations” are expressed and examined. After selecting the relevant output, the detection operation is performed on it. With the detected output in hand, the error detection process can be performed on it. As mentioned in Section 4, three types of errors are considered for the simulator.

- Short circuit current decrease caused by partial shadow (e1)
- Output voltage error caused by partial shadow (e2)
- Damage to solar cell array making low voltage and low power (e3)

In order to create a suitable and comprehensive database of the error intensity pattern in the face of random perturbations with any variance and each level of error we assume the nominal error is N_0. Scale of error intensity pattern in databases is considered as (−20%, −10%, 0.0%, +10%, +20%) × N_0. The values expressed as a percentage can be varied according to the type of process and the allowable range. Form (V.LOW, LOW, NORMAL, MEDIUME, HIGH) is defined. With five levels of error and three specific error scenarios, the simulation will be performed five times for each error. In this simulator, four sensors are used for sampling. The number of signals stored in the library will be five signals per sensor at each error level. The number of signals that will be processed in the error detection strategy to perform the simulation is equal to 4 × 3 × 5 = 60. Therefore, with five signals per sensor at different error levels, the trend extraction algorithm starts extracting the initial patterns from the signals and, respectively, by running one of the errors defined for the turbine, a sequence of the initial patterns including the number and length of the segment. The first and second derivative symbols for each segment and column of the original patterns are stored in libraries according to Table 3, which will be ready and available in all three signaling errors, taking into account the execution of all three e1, e2 and e3 errors. By performing a fuzzy algorithm and measuring the similarity between the unknown signal patterns and the database patterns, the degree of close similarity with the database data will indicate the occurrence of the relevant error.

### 5.1. Occurrence of Partial Shadow

**Figure 9:**
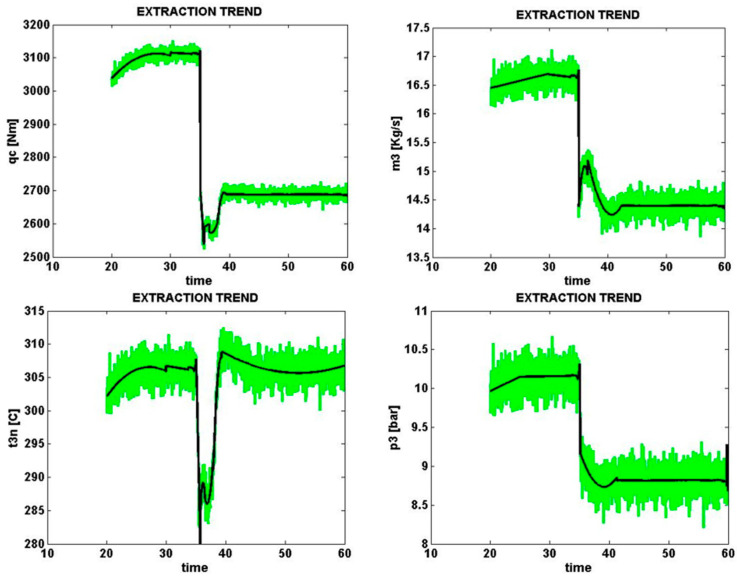
Occurrence of partial shadow to zero error rate (normal).

**Figure 10:**
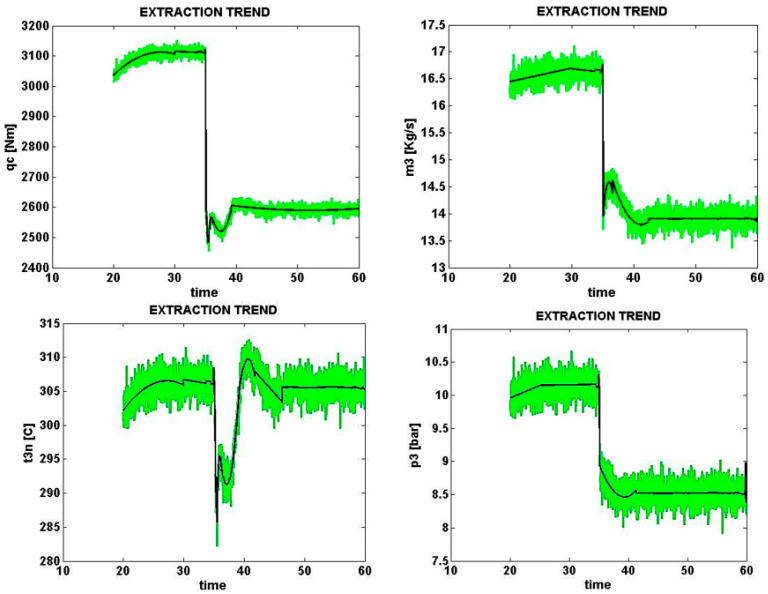
Occurrence of partial shadow amounting to +0.2% error.

Signals sampled from four different parts of the solar cell with an error of e1 (dirty occurrence of partial shadow) with a normal level of zero percent are shown in Figure 9. The same condition with a high error level (+20%) is shown in Figure 10.

Solar cell loss power *q_c* is shown from 4 sampled signals taking into account all error levels in the general form as $qc_{e1}^{-20},qc_{e1}^{-10},qc_{e1}^{0.0},qc_{e1}^{+10},qc_{e1}^{+20}.$ The results show that at the time of error e1 by zero and 20%, in accordance with the above discussion and the data of the tables, in both cases the value obtained compared to the other components of the same column is the maximum value of 1.0. This means it is the closest and most similar to the e1 error pattern.

### 5.2. Output Voltage Error Caused by Partial Shadow (e2)

**Figure 11:**
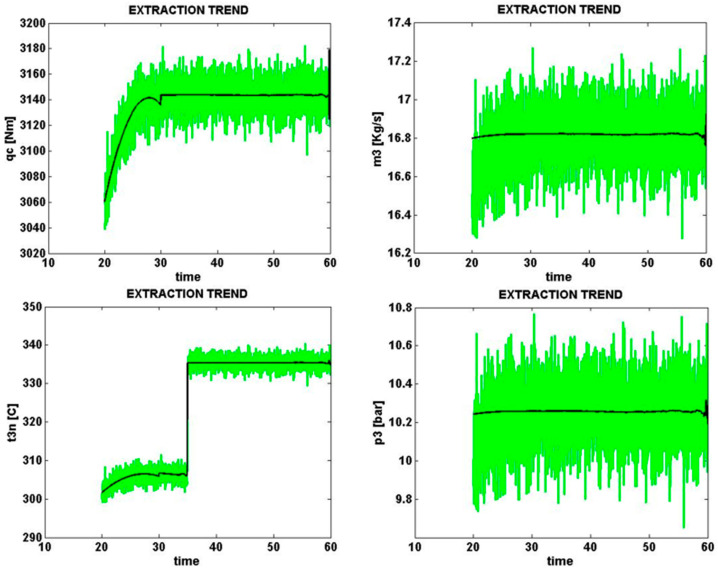
Error caused by a fault in the output voltage due to the occurrence of partial shadow, zero error rate (normal).

**Figure 12:**
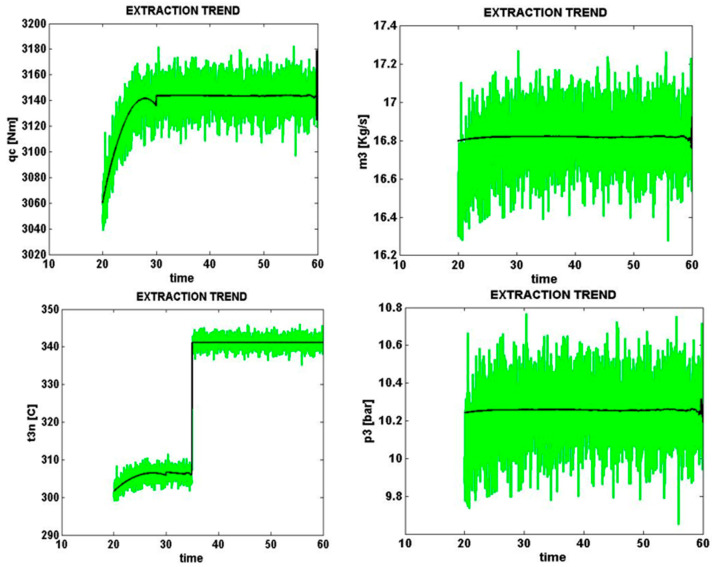
Error caused by a fault in the output voltage due to the occurrence of partial shadow by 20% error.

Signals sampled from four different parts of the solar cell with an e2 error (thermocouple sensor error) and a normal level of 0% are shown in Figure 11 similar to the same condition with a high error level (+20%) in Figure 11 and Figure 12. For example, taking into account all error levels, the signal t_3n_ is the temperature of the fluid leaving the compressor in the general form $t_{3n}{\;}_{e2}^{-20},t_{3n}{\;}_{e2}^{-10},t_{3n}{\;}_{e2}^{0.0},t_{3n}{\;}_{e2}^{+10},t_{3n}{\;}_{e2}^{+20}$. In this case, due to the general similarity of the signals in all cases, the maximum values of the similarity and the maximum limit of the dominant signals is 1.0. We will need to select other voltage outputs so that it can respond logically to various errors.

### 5.3. Solar Cell Damage and Lack of Partial Shade

**Figure 13:**
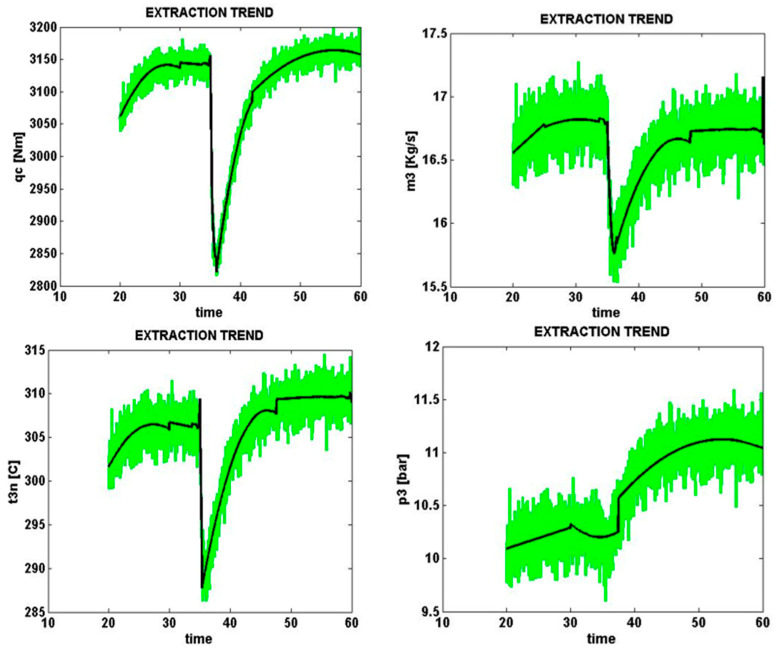
Error caused by solar cell damage, zero percent error (normal).

Solar cell signals sampled from four different parts of the solar cell with an e3 error (solar cell damage) with a normal level of zero percent are shown in Figure 13. The same condition with a high error level (+20%) is shown in Figure 13.

Figure 13 shows the error caused by solar cell damage, the presence of cracks in the cell surface, and the reduction of the production power level by 20% of the total power.

The speed of error detection depends on how it is executed and the type of methods that have been used. The speed of execution in the solar cell fault detection algorithm by event analysis method is very important for sensitive industries such as electricity and power plants, regardless of considering “offline” data recording times. For this purpose, increasing the speed and reducing the computation time as well as detecting multiple faults in the time series obtained from the sensors are the features of a successful algorithm. This is also one of the cases that can be considered for the correction of many methods and improvement upon the computational speed to an acceptable extent. In the field of noise estimation, which is one of the most important issues in detection, the use of robust and reliable methods can increase the certainty of detection and reduce the potential for detection. For example, it can benefit from relatively new topics in the field of estimation, such as the use of wavelet transforms and be considered as a safe method.

It is necessary to enrich the techniques of using quantitative analysis in addition to the qualitative analysis discussed in the fuzzy discussion. Such an enrichment could be effective in promoting and furthering decision-making power. This, in itself, requires the addition of research in this area. As an example, we can refer to the moment of error. Identifying the moment of occurrence of the error is undertaken in this article by considering the time of occurrence manually and defined. Moreover, to have instantaneous error detection and measurement of time and moment of occurrence, it is necessary to add other algorithms that can be considered in a comprehensive plan as a separate project with detailed content. However, it is possible to identify the moment of occurrence by having parameters related to the signal, such as signal amplitude, amplitude, error rate, and so on. This, as an efficient secondary tool, helps in error detection in the field of quantitative analysis. The use of neural methods in the last loop of the detection algorithm can contribute to the separation of the type and severity of the error in combination with fuzzy logic and play a valuable role in achieving a definite and reliable answer. Is a tool which will improve the detection speed. Due to the transparency and comprehensibility of the event analysis algorithm, the field for operators and engineers to enter the optimization factors in this field is wide. The components of the error detection algorithm can be integrated, but, at the same time, the distances between the components are easily distinguishable. The algorithm can be fragmented and defined by optimization methods, and algorithm components can be improved to increase analytical power, detection certainty, and speed. It is hoped that in the future, various new methods will be used to optimize the algorithm, and we will witness significant advances in error detection.

## 6. Conclusions

In most of the proposed methods to detect the type of error in photovoltaic systems, changes in the amount of output power and changes in voltage-current curve have been used to determine the type of error. The effect that the occurrence of a small shadow on the power and voltage-current curve is in practice very similar to the effects that different errors have on the power and voltage-current curve. According to studies in the field of pattern recognition using intelligent methods, the type of input and its volume have a high impact on the accuracy of detection. New features of voltage-current curve and power changes can be extracted and used as a classifier input to improve detection accuracy. Due to the importance of online fault detection and its type detection in photovoltaic systems, in this study, a new method has been proposed for this purpose. The proposed method will consist of three parts: the part for detecting the occurrence of perturbation (partial shadow and inherent error of the solar cell), the part for separating the errors, and the part for detecting the type of error. The studied intelligent method can be a method based on intelligent algorithms and algorithms of fuzzy structures.

## Figures and Tables

**Table 1:** List of errors checked (NT-R5E3E PV module) [24].

Abbreviation	Error Type
PF1	Normal type
PF2	Short circuit two modules to each other from two different arrays
PF3	Short-circuiting two modules of an array when the two arrays are connected in series.
PF4	Short-circuiting three modules of an array when the two arrays are connected in series.
PF5	Short-circuit a module to ground from an array when the two arrays are connected in series.
PF6	Short-circuiting two modules to ground from two arrays while the two arrays are connected in series.
PF7	Short-circuiting three modules to ground from two arrays while the two arrays are connected in series.
PF8	Short-circuiting two modules to ground from one array and one module from another array when the two arrays are connected in series.
PF9	Short-circuit three modules to ground from one array and one module from another array when the two arrays are connected in series.
PF10	Short-circuit three modules to ground from one array and one module from another array when the two arrays are connected in series.

**Table 2:** $S_{\;p_{i}\;p_{j}}$ Similarity matrix.

A	B	C	D	E	F	G
A	1	0	0.25	0	0.25	0
B	0	1	0.75	0	0	0
C	0.25	0.75	1	0	0	0
D	0	0.5	0.75	0	0	0
E	0	0	0	1	0.75	0.5
F	0.25	0	0	0.75	1	0.75
G	0	0	0	0.5	0.25	1

**Table 3:** Sample of data stored in the library.

Segment Number	Segment Length	First Derivative	Second Derivative	Name of the Original Pattern
1	833	0.083626	−4105.83	‘D’
2	312	−0.01182	0	‘F’
3	68	−0.01255	0	‘F’
4	33	0.034637	0	‘C’
5	4	0.295438	0	‘C’
6	2	−428.917	0	‘F’
7	16	−0.6471	0	‘F’
8	16	−2.4092	0	‘F’
9	32	−1.9283	4409387	‘E’
10	63	0.149232	0	‘C’
11	122	0.246181	94192.44	‘B’
12	114	0.710967	−237422	‘D’

## Data Availability

Not applicable.

## References

[1] O’Neill S. (2020). Global CO_2_ emissions level off in 2019, with a drop predicted in 2020. Engineering.

[2] Nasirpour M.H., Sharifi A., Ahmadi M., Jafarzadeh Ghoushchi S. (2021). Revealing the relationship between solar activity and COVID-19 and forecasting of possible future viruses using multi-step autoregression (MSAR). Environ. Sci. Pollut. Res..

[3] Jafarzadeh-Ghoushchi S., Sharifi A., Ahmadi M., Maghami M. (2017). Statistical study of seasonal storage solar system usage in Iran. J. Sol. Energy Res..

[4] Wang S., Tarroja B., Schell L.S., Shaffer B., Samuelsen S. (2019). Prioritizing among the end uses of excess renewable energy for cost-effective greenhouse gas emission reductions. Appl. Energy.

[5] Liu L., Li H., Xue Y., Liu W. (2014). Reactive power compensation and optimization strategy for grid-interactive cascaded photovoltaic systems. IEEE Trans. Power Electron..

[6] Ghoushchi S.J., Manjili S., Mardani A., Saraji M.K. (2021). An extended new approach for forecasting short-term wind power using modified fuzzy wavelet neural network: A case study in wind power plant. Energy.

[7] Gielen D., Boshell F., Saygin D., Bazilian M.D., Wagner N., Gorini R. (2019). The role of renewable energy in the global energy transformation. Energy Strategy Rev..

[8] Villegas-Mier C.G., Rodriguez-Resendiz J., Álvarez-Alvarado J.M., Rodriguez-Resendiz H., Herrera-Navarro A.M., Rodríguez-Abreo O. (2021). Artificial neural networks in MPPT algorithms for optimization of photovoltaic power systems: A review. Micromachines.

[9] Jafarzadeh Ghoushchi S., Ab Rahman M.N., Raeisi D., Osgooei E., Jafarzadeh Ghoushji M. (2020). Integrated decision-making approach based on SWARA and GRA methods for the prioritization of failures in solar panel systems under Z-information. Symmetry.

[10] Rokonuzzaman M., Mishu M.K., Amin N., Nadarajah M., Roy R.B., Rahman K.S., Buhari A.M., Binzaid S., Shakeri M., Pasupuleti J. (2021). Self-Sustained autonomous wireless sensor network with integrated solar photovoltaic system for internet of smart home-building (IoSHB) applications. Micromachines.

[11] Zhang X., Huang C., Wang L., Zhou M. (2018). Collaborative R&D between multicrystalline silicon ingots and battery efficiency improvement—Effect of shadow area in multicrystalline silicon ingots on cell efficiency. J. Semicond..

[12] Li M., Deng H., Zhang Y., Li K., Huang S., Liu X. (2020). Ultra-low frequency eccentric pendulum-based electromagnetic vibrational energy harvester. Micromachines.

[13] Refaat S.S., Abu-Rub H., Sanfilippo A.P., Mohamed A. (2018). Impact of grid-tied large-scale photovoltaic system on dynamic voltage stability of electric power grids. IET Renew. Power Gener..

[14] Al-Shetwi A.Q., Sujod M.Z., Blaabjerg F., Yang Y. (2019). Fault ride-through control of grid-connected photovoltaic power plants: A review. Sol. Energy.

[15] Salim K., Asif M., Ali F., Armghan A., Ullah N., Mohammad A.-S., Al Ahmadi A.A. (2022). Low-Stress and Optimum Design of Boost Converter for Renewable Energy Systems. Micromachines.

[16] Diaz-Saldierna L.H., Leyva-Ramos J. (2021). High Step-Up Converter Based on Non-Series Energy Transfer Structure for Renewable Power Applications. Micromachines.

[17] Hafeznia H., Yousefi H., Astaraei F.R. (2017). A novel framework for the potential assessment of utility-scale photovoltaic solar energy, application to eastern Iran. Energy Convers. Manag..

[18] Teshome D., Lee C., Lin Y., Lian K. (2016). A modified firefly algorithm for photovoltaic maximum power point tracking control under partial shading. IEEE J. Emerg. Sel. Top. Power Electron..

[19] Sajadian S., Ahmadi R. (2017). Distributed maximum power point tracking using model predictive control for photovoltaic energy harvesting architectures based on cascaded power optimizers. IEEE J. Photovolt..

[20] Silverman T.J., Deceglie M.G., Subedi I., Podraza N.J., Slauch I.M., Ferry V.E., Repins I. (2018). Reducing operating temperature in photovoltaic modules. IEEE J. Photovolt..

[21] Yousri D., Allam D., Eteiba M., Suganthan P.N. (2019). Static and dynamic photovoltaic models’ parameters identification using chaotic heterogeneous comprehensive learning particle swarm optimizer variants. Energy Convers. Manag..

[22] Harrou F., Taghezouit B., Sun Y. (2019). Robust and flexible strategy for fault detection in grid-connected photovoltaic systems. Energy Convers. Manag..

[23] Kaid I.E., Hafaifa A., Guemana M., Hadroug N., Kouzou A., Mazouz L. (2018). Photovoltaic system failure diagnosis based on adaptive neuro fuzzy inference approach: South Algeria solar power plant. J. Clean. Prod..

[24] Harrou F., Sun Y., Taghezouit B., Saidi A., Hamlati M.-E. (2018). Reliable fault detection and diagnosis of photovoltaic systems based on statistical monitoring approaches. Renew. Energy.

[25] Kalam A., Runjhun R., Mahapatra A., Tavakoli M.M., Trivedi S., Tavakoli Dastjerdi H., Kumar P., Lewiński J., Pandey M., Prochowicz D. (2020). Interpretation of resistance, capacitance, defect density, and activation energy levels in single-crystalline MAPbI3. J. Phys. Chem. C.

[26] Trivedi S., Prochowicz D., Parikh N., Mahapatra A., Pandey M.K., Kalam A., Tavakoli M.M., Yadav P. (2021). Recent progress in growth of single-crystal perovskites for photovoltaic applications. ACS Omega.

[27] Benaicha M., Dehimi L., Pezzimenti F., Bouzid F. (2020). Simulation analysis of a high efficiency GaInP/Si multijunction solar cell. J. Semicond..

[28] Pilakkat D., Kanthalakshmi S. (2019). An improved P&O algorithm integrated with artificial bee colony for photovoltaic systems under partial shading conditions. Sol. Energy.

[29] Chao K.-H., Chen C.-T. (2017). A remote supervision fault diagnosis meter for photovoltaic power generation systems. Measurement.

[30] Zamani M.A., Yazdani A., Sidhu T.S. (2012). A communication-assisted protection strategy for inverter-based medium-voltage microgrids. IEEE Trans. Smart Grid.

[31] Saleh K.A., El-Saadany E.F., Zeineldin H. Current-Based Protection Scheme for Faults Within Utility-Scale Photovoltaic Arrays. Proceedings of the 2018 IEEE Electrical Power and Energy Conference (EPEC).

[32] González-Serrano F.-J., Navia-Vázquez Á., Amor-Martín A. (2017). Training support vector machines with privacy-protected data. Pattern Recognit..

[33] Alaíz C.M., Suykens J.A. (2018). Modified Frank–Wolfe algorithm for enhanced sparsity in support vector machine classifiers. Neurocomputing.

[34] Gholami R., Fakhari N. (2017). Support vector machine: Principles, parameters, and applications. Handbook of Neural Computation.

